# Factors associated with readmissions in psychiatric inpatient care: a prospective cohort study based on hospital registers

**DOI:** 10.1186/s12888-024-06193-1

**Published:** 2024-10-25

**Authors:** Marianna Virtanen, Laura Peutere, Mikko Härmä, Annina Ropponen

**Affiliations:** 1 School of Educational Sciences and Psychology, University of Eastern Finland, P.O. Box 111, Joensuu, 80101 Finland; 2 Research Unit, Social Insurance Institution of Finland (KELA), P.O. Box 450, Helsinki, 00056 Finland; 3 Finnish Institute of Occupational Health, P.O. Box 40, Työterveyslaitos, 00032 Finland; 4 Division of Insurance Medicine, Department of Clinical Neuroscience, Karolinska Institutet, Stockholm, 17177 Sweden

**Keywords:** Healthcare, Psychiatric care, Readmission, Register study

## Abstract

**Background:**

Readmissions in psychiatric inpatient care may reflect problems in the provision of care, but the underlying factors are not well known. We examined the associations of patient characteristics (sociodemographic factors, diagnoses), treatment (duration, previous episodes, neuromodulation) and ward overload with psychiatric inpatient readmissions and multiple readmissions in Finland.

**Methods:**

We used a routinely collected data pool from one hospital district and followed all 2052 hospitalizations that started in 2018. The outcomes were readmission within 30 days and one year, and among those with readmission, the number of readmissions.

**Results:**

Of the patients, 11% had readmission within 30 days and 33% had readmission within one year. Women, those with previous hospitalizations, those with an ICD-10 diagnosis from the ‘behavioural syndromes associated with physiological disturbances and physical factors / disorders of adult personality and behaviour’ group, those with a diagnosis from the ‘neurotic, stress-related and somatoform disorders’ group, those with psychotic disorders, and those who received neuromodulation treatment were more likely to have readmissions. Having a diagnosis of ‘disorders of psychological development’ or ‘behavioural and emotional disorders with onset usually occurring in childhood and adolescence’ was associated with a lower likelihood of readmission. The duration of treatment and ward overload during the index period were not associated with readmission.

**Conclusions:**

The findings of this study suggest possible risk factors for readmission and can be used to plan psychiatric care. To some degree, the risk factors varied between different readmission types. It is important to examine whether there are unmet treatment needs in psychiatric inpatient care for children and adolescents.

**Supplementary Information:**

The online version contains supplementary material available at 10.1186/s12888-024-06193-1.

## Background

Several reforms in psychiatric inpatient care have occurred in recent decades. The changes that have taken place are characterized by a strong reduction in inpatient beds and an emphasis on outpatient care. Consequently, in 2021, there were 2700 hospital beds in psychiatric hospitals in Finland, which was 41% less than in 2015 and 88% less than in 1970 [1]. There are some issues in the current situation, as patients who need acute care are often discharged too early, which may result in rapidly recurring admissions [2]. In Finland, approximately 10% of patients return to psychiatric inpatient care within 30 days, which is an average proportion in some international comparisons [3, 4].

Recurrent psychiatric admissions are not well understood, although it has been suggested that they may involve some adverse effects [5, 6]. At the hospital level, recurrence is an internationally agreed measure of the quality of care [6] and is also recommended for use in Finland [4]. The adverse effects of recurrent admissions include, for example, the deterioration of quality of life caused by the interruption of everyday routines and interruptions in outpatient care and support networks [5, 7] and the risk of feeling insecure in inpatient care [8]. However, recurrent admissions can also reflect the nature of the disease and be part of the treatment, so they cannot be considered unequivocally good or bad [9]. For example, in a focus group interview study carried out in six European countries (including Finland), readmissions were sometimes experienced as a relief or part of recovery [6]. On the other hand, readmissions can reflect the quality of the treatment received and whether the patient has sufficient support in outpatient care [10].

There are systematic reviews that have collected studies concerning psychiatric readmissions. The findings suggest that the number of previous admissions is a strong predictor of new admissions [10, 11], as well as the duration of previous episodes (both longer and shorter) [10, 12]; a psychotic-level mental disorder, such as schizophrenia [10, 11]; comorbid somatic diseases [13]; and some sociodemographic factors, such as the patient’s low socioeconomic position [10, 11]. One review focused on system-level factors, such as regional differences, which have rarely been examined in this field [12]. Few studies have examined organization-level factors, such as personnel resources, and the findings have been mixed [12].

Recent research findings confirm previous observations on patient-related factors that are associated with readmission [3, 9, 14–17]. Of those, a study targeting hospitals located in Beijing, China, had quite a similar setup to our study, but ward overload was not the subject of the study [14]. In an Italian study, the number of hospital beds was not associated with readmission, although a larger number of personnel in the hospital district was associated with a lower probability of readmission [9]. A US hospital- and system-level analysis reported that the size of the hospital, ownership type (public/private), availability of outpatient care, teaching hospital status, area-level income, and area-level number of mental health personnel per resident were not associated with readmission [15]. A Japanese study compared different types of units and found that readmission was less likely in units with greater personnel resources [16]. Similarly, a study carried out in South Korea revealed that more patients per nurse in the unit were associated with readmissions [17]. Only a handful of studies have been conducted in Finland. One study reported that there were great differences in readmission rates between hospital districts [5]. Of the patients, 8% did not have outpatient contacts after discharge, and among them, the risk of readmission was greatest [5]. According to an international project, younger age, shorter duration of admission, psychotic-level disorders, and somatic comorbidities predicted a new treatment period [3].

The gaps in previous research include the lack of focus on organisational factors, such as ward overload, and in addition, few studies have considered repeated readmissions. This is an important point, as there might be differences in the predictors of one versus repeated readmissions [18]. Furthermore, the association between receiving treatments and readmission has rarely been examined. In the psychiatric setting, these include, for example, neuromodulation treatment, such as electroconvulsive therapy (ECT), which has been found to be associated with a lower risk of readmission [14]. Finally, interactions detecting vulnerable populations for the effect or ward overload on readmission, such as those with comorbid mental disorders, have not been examined. It is also not known whether women and men in different diagnostic groups are more prone to experience readmission.

The aims of this study were to examine the following:

1. Which patient-, treatment- and ward-level factors are associated with psychiatric readmissions?
2. Are there different predictors when the focus is on one versus repeated readmissions?
3. Are there interactions between ward overload and psychiatric comorbidity predicting readmission?
4. Are there interactions between sex and psychiatric diagnoses predicting readmission?

## Methods

### Sample

The data for this study were derived from the patient data pool of Auria, Finland, covering individual-level patient data and administrative data for 2018–2020 in one hospital district. The data pool is a centralized system that collects all the data from one hospital district, irrespective of the format. We obtained permission for its use for research purposes. These types of secondary data do not require an ethical assessment. We analysed the data in an anonymized format. For this study, we selected data from all patients admitted to psychiatric inpatient wards whose hospitalization period began in 2018 (Jan 1 2018 – Dec 31 2018, *n* = 2052). For each patient, we selected the first admission during this period to represent the index period. If the patient was newly admitted immediately after the end of the index period (within the next calendar day at the latest), the two or more admissions were merged into one. In the regression analyses, we included only patients who were alive during the entire follow-up (*n* = 18 died within 30 days after discharge, and *n* = 66 died within one year after discharge).

### Patient-, treatment- and ward-level factors

All patient and hospital characteristics were assessed at the beginning of the index admission. The patients’ sociodemographic characteristics included age and sex. For the clinical data, we formulated dichotomous variables from all diagnoses of mental disorders (yes/no). All diagnoses (main and side diagnoses) were considered. Diagnoses of mental disorders were grouped according to the International Classification of Diseases, 10th Revision (ICD-10) diagnosis codes as follows: mental and behavioural disorders due to psychoactive substance use (F10–F19); psychotic disorders (schizophrenia, schizotypal and delusional disorders, manic episode, bipolar affective disorder, F20–F29, F30–F31); depressive and other mood (affective) disorders (F32–F39); neurotic, stress-related and somatoform disorders (F40–F48); behavioural syndromes associated with physiological disturbances and physical factors; disorders of adult personality and behaviour (F50–F59, F60–F69); disorders of psychological development; behavioural and emotional disorders with onset usually occurring in childhood and adolescence (F80–F89, F90–F98). We also calculated the number of psychiatric diagnoses groups described above and categorized them into two groups: 0–1 and 2 or more diagnoses (there were 86 patients, i.e., 4% without a psychiatric diagnosis). In addition, we formulated a dichotomous variable based on whether the patient had any diagnosis of somatic disease (yes/no), as has been done previously [3].

Of the available data on treatments, we considered neuromodulation treatment (e.g., ECT, transcranial serial magnetic stimulation treatment). In addition, we calculated the duration of hospital stay and the number of previous admissions for which the data were available since 2008.

Bed occupancy in each ward represented hospital ward overload. This was calculated for each of the 7 hospital wards for every calendar day between 2018 and 2020. We included the admission day as the number of patients but not the discharge day. Overload was denoted if the number of patients exceeded the median number of patients. The median ward overload was calculated for each ward across the entire calendar year. We further determined how many days on average the patient was exposed to ward overload during his/her stay (this was calculated for the index admission). This information was further divided as follows: <20%, 20–80% and > 80% of the days. The corresponding measure has been used in previous studies, with the aim of examining, e.g., nurse staffing levels and their associations with patient outcomes [19, 20].

### Readmission

The outcomes included (1) readmission within 30 days, (2) readmission within one year after the end of the index admission, and (3) the number of readmissions the patient had during the following year after the end of the index admission. The first two outcomes are the most used indicators of readmission in the international literature [3, 10, 14], whereas the number of readmissions has rarely been examined [18]. For the third outcome, we included only patients with at least one readmission. The number of readmissions was divided into two groups: two or more readmissions versus one.

### Statistical analyses

We first provided descriptive statistics of the patients. Then, we conducted logistic regression analysis to examine the associations between patient-, treatment- and ward-related predictors and readmission outcomes and reported odds ratios (ORs) and their 95% confidence intervals (CIs) for readmission within 30 days, readmission within a year, and two or more readmissions versus one. To examine robustness of the results, we conducted a total of four different models: (1) univariate associations; (2) a model where all predictors were entered in the same model; (3) a model where only individual-level factors were included; (4) a model where individual and treatment factors were included. We also analysed interactions between ward overload and psychiatric comorbidity and between sex and psychiatric diagnoses, by entering the interaction term (e.g., sex*diagnosis) into the model that included age and the main effects of sex and diagnosis.

## Results

There were a total of 2052 new treatment episodes in 2018. Of the patients, 52% were women, and 60% were 16–50 years of age (Supplementary Table 1). The most prevalent diagnoses were psychotic disorders (F20–F29, F30–F31, 43%) and depressive and other mood disorders (F32–F39, 35%). The majority (68%) had a mental disorder only from one of the six categories, 10% had somatic diseases, and 2% had received neuromodulation treatment. Of the treatment periods, 28% lasted 1–6 days, and half of them lasted 7–29 days. Most of the patients (64%) did not have any history of psychiatric hospitalization in the hospital district under study. Of the patients, 29% spent their hospital stay in a ward where more than 80% of the days during their treatment period were indicated as being highly occupied. Of the patients, 11% had a readmission within 30 days, and 33% had a readmission within a year. Of those who had at least one readmission (*n* = 662), most (58%) had one readmission, and 42% had two or more readmissions within a year.

Table 1 examines the associations of different factors with readmission within 30 days. Age, sex, psychotic disorder, depression, substance use disorder, somatic disease, and duration of hospitalization were not associated with readmission; one exception was 30–89 days being associated with lower risk in the second model. Among the diagnostic groups, neurotic, stress-related and somatoform disorders (F40–F48) were associated with readmission in both models. The group including behavioural syndromes associated with physiological disturbances and physical factors (F50–F59) and disorders of adult personality and behaviour (F60–F69) was associated with readmission (in the multivariate model only). Disorders of psychological development and behavioural and emotional disorders with onset usually occurring in childhood and adolescence (F80–F89, F90–F98) were associated with a lower likelihood of readmission (in the univariate model only). Neuromodulation treatment was associated with a greater likelihood, but comorbid mental disorder was associated with a lower likelihood of readmission (in the multivariate model only). Compared with the patients who did not have a history of psychiatric hospitalization, those with two and three or more previous hospitalization had a greater likelihood of readmission in both models. Having comorbid mental disorders was associated with a lower likelihood of readmission (in the multivariate model only). Ward overload during the index period was not associated with readmission.

**Table 1:** Association of patient-, treatment- and ward-level characteristics with readmission within 30 days (*N* = 2034)

Characteristic	Univariate		Multivariate^a^		
	OR	95% CI		OR	95% CI
Sex					
Female	1.00			1.00	
Male	0.83	0.63–1.11		1.00	0.74–1.35
Age group					
0–15	1.00			1.00	
16–30	1.28	0.79–2.09		0.90	0.52–1.57
31–50	0.94	0.56–1.57		0.67	0.37–1.23
51–70	1.18	0.69–2.02		0.83	0.45–1.56
71–	0.98	0.52–1.85		0.70	0.35–1.42
Mental and behavioural disorders due to psychoactive substance use (F10–F19): no	1.00			1.00	
yes	0.73	0.48–1.12		1.00	0.56–1.77
Psychotic disorders; Schizophrenia, schizotypal and delusional disorders (F20–F29), manic episode, bipolar affective disorder (F30–F31): no	1.00			1.00	
yes	0.92	0.69–1.22		1.44	0.85–2.44
Depressive disorders and other mood disorders (F32–F39): no	1.00			1.00	
yes	1.21	0.91–1.62		1.59	0.94–2.67
Neurotic, stress-related and somatoform disorders (F40–F48): no	1.00			1.00	
yes	1.41	1.02–1.95		2.10	1.21–3.62
Behavioural syndromes associated with physiological disturbances and physical factors (F50–F59), disorders of adult personality and behaviour (F60–F69): no	1.00			1.00	
yes	1.45	0.96–2.19		1.87	1.06–3.31
Disorders of psychological development (F80–F89), behavioural and emotional disorders with onset usually occurring in childhood and adolescence (F90–F98): no	1.00			1.00	
yes	0.50	0.27–0.91		0.59	0.27–1.26
Number of mental disorder in the examined categories					
0–1	1.00			1.00	
2+	0.98	0.72–1.32		0.51	0.27–0.98
Somatic disease: no	1.00			1.00	
yes	0.92	0.57–1.50		0.99	0.60–1.63
Neuromodulation treatment: no	1.00			1.00	
yes	2.08	0.99–4.37		2.41	1.08–5.36
Length of index treatment period (days): 1–6	1.00			1.00	
7–29	0.92	0.67–1.28		0.86	0.61–1.20
30–89	0.72	0.46–1.11		0.59	0.37–0.95
90 or more	0.67	0.28–1.61		0.48	0.19–1.22
Number of previous hospitalizations: 0	1.00			1.00	
1	1.34	0.91–1.99		1.47	0.98–2.20
2	2.01	1.24–3.23		2.08	1.27–3.39
3 or more	1.53	1.03–2.27		1.61	1.07–2.44
Exposure to ward overload during index treatment period: <20% of days	1.00			1.00	
20–80% of days	0.94	0.67–1.33		1.10	0.77–1.58
> 80% of days	0.94	0.65–1.36		0.93	0.64–1.36

^a^All variables entered simultaneously

OR = odds ratio; CI = confidence interval

The corresponding associations for readmission within one year are presented in Table 2. Men were less likely than women to return to treatment, and those with a psychotic disorder (F20–F29, F30–F31) were more likely to have a readmission in both models. The oldest age group (> 70 years) had a lower likelihood of readmission in the multivariate model. The diagnostic group including behavioural syndromes associated with physiological disturbances and physical factors or disorders of adult personality and behaviour (F50–F59, F60–F69) was also associated with readmission in both models. Having a somatic disease was associated with a lower likelihood of readmission in the univariate model but no longer in the multivariate model. A history of psychiatric hospitalization was associated with readmission. The rest of the diagnostic groups, comorbid mental disorders, neuromodulation treatment, duration of the index treatment period, and ward overload were not associated with readmission within a year.

**Table 2:** Association of patient-, treatment- and ward-level characteristics with readmission within one year (*N* = 1986)

Characteristic	Univariate		Multivariate^a^		
	OR	95% CI		OR	95% CI
Sex					
Female	1.00			1.00	
Male	0.79	0.65–0.95		0.80	0.65–0.98
Age group					
0–15	1.00			1.00	
16–30	1.26	0.92–1.73		0.96	0.66–1.39
31–50	1.05	0.76–1.47		0.75	0.50–1.13
51–70	1.12	0.79–1.59		0.81	0.53–1.24
71–	0.69	0.44–1.07		0.54	0.33–0.90
Mental and behavioural disorders due to psychoactive substance use (F10–F19): no	1.00			1.00	
yes	1.01	0.78–1.30		1.34	0.91–1.96
Psychotic disorders; Schizophrenia, schizotypal and delusional disorders (F20–F29), manic episode, bipolar affective disorder (F30–F31): no	1.00			1.00	
yes	1.22	1.01–1.47		1.46	1.01–2.10
Depressive disorders and other mood disorders (F32–F39): no	1.00			1.00	
yes	0.90	0.74–1.09		1.18	0.82–1.69
Neurotic, stress-related and somatoform disorders (F40–F48): no	1.00			1.00	
yes	1.04	0.83–1.31		1.43	0.97–2.10
Behavioural syndromes associated with physiological disturbances and physical factors (F50–F59), disorders of adult personality and behaviour (F60–F69): no	1.00			1.00	
yes	1.70	1.27–2.26		1.80	1.21–2.68
Disorders of psychological development (F80–F89), behavioural and emotional disorders with onset usually occurring in childhood and adolescence (F90–F98): no	1.00			1.00	
yes	0.84	0.62–1.16		1.00	0.63–1.58
Number of mental disorders in the examined categories					
0–1	1.00			1.00	
2+	1.09	0.90–1.33		0.67	0.44–1.05
Somatic disease: no	1.00			1.00	
yes	0.69	0.49–0.96		0.72	0.51–1.03
Neuromodulation treatment: no	1.00			1.00	
yes	1.62	0.89–2.94		1.64	0.87–3.09
Length of index treatment period (days): 1–6	1.00			1.00	
7–29	1.02	0.82–1.28		1.02	0.80–1.28
30–89	1.13	0.85–1.49		1.05	0.77–1.43
90 or more	1.38	0.84–2.28		1.10	0.64–1.90
Number of previous hospitalizations: 0	1.00			1.00	
1	1.43	1.09–1.86		1.41	1.07–1.85
2	2.36	1.66–3.36		2.31	1.61–3.30
3 or more	2.40	1.84–3.15		2.31	1.74–3.05
Exposure to ward overload during index treatment period: <20% of days	1.00			1.00	
20–80% of days	1.05	0.84–1.32		1.06	0.83–1.36
> 80% of days	0.87	0.68–1.12		0.85	0.65–1.10

^a^All variables entered simultaneously

OR = odds ratio; CI = confidence interval

Table 3 presents the corresponding associations with the number of readmissions among those patients with at least one readmission. Disorders of psychological development and behavioural and emotional disorders with onset usually occurring in childhood and adolescence (F80–F89, F90–F98) were associated with a lower likelihood of having two or more readmissions compared to one readmission in both models. In addition, neuromodulation treatment was associated with multiple readmissions. Having a history of psychiatric hospitalization was associated with the number of readmissions in a dose‒response manner; the more previous hospitalizations the patient had, the more likely there were at least two readmissions. The rest of the diagnostic groups, age, sex, somatic disease, length of the treatment period and ward overload were not associated with the number of readmissions.

**Table 3:** Association of patient-, treatment- and ward-level characteristics with multiple readmissions versus one readmission within a year (*N* = 662)

Characteristic	Univariate			Multivariate^a^		
	OR	95% CI		OR	95% CI	
Sex						
Female	1.00			1.00		
Male	0.84	0.61–1.14		0.87	0.62–1.24	
Age group						
0–15	1.00			1.00		
16–30	1.25	0.73–2.13		0.72	0.37–1.37	
31–50	1.37	0.78–2.39		0.73	0.36–1.46	
51–70	1.32	0.73–2.38		0.72	0.35–1.50	
71–	0.93	0.43–2.05		0.48	0.19–1.21	
Mental and behavioural disorders due to psychoactive substance use (F10–F19): no	1.00			1.00		
yes	1.20	0.79–1.82		1.02	0.55–1.88	
Psychotic disorders; Schizophrenia, schizotypal and delusional disorders (F20–F29), manic episode, bipolar affective disorder (F30–F31): no	1.00			1.00		
yes	1.04	0.76–1.41		0.87	0.47–1.58	
Depressive disorders and other mood disorders (F32–F39): no	1.00			1.00		
yes	0.97	0.70–1.35		0.71	0.39–1.29	
Neurotic, stress-related and somatoform disorders (F40–F48): no	1.00			1.00		
yes	1.08	0.74–1.58		1.05	0.56–1.96	
Behavioural syndromes associated with physiological disturbances and physical factors (F50–F59), disorders of adult personality and behaviour (F60–F69): no	1.00			1.00		
yes	1.53	0.99–2.37		1.13	0.61–2.09	
Disorders of psychological development (F80–F89), behavioural and emotional disorders with onset usually occurring in childhood and adolescence (F90–F98): no	1.00			1.00		
yes	0.51	0.29–0.92		0.36	0.16–0.80	
Number of mental disorders in the examined categories						
0–1	1.00			1.00		
2+	1.19	0.86–1.65		1.28	0.63–2.61	
Somatic disease: no	1.00			1.00		
yes	1.22	0.68–2.17		1.10	0.59–2.05	
Neuromodulation treatment: no	1.00			1.00		
yes	4.41	1.58–12.28		5.35	1.73–16.55	
Length of index treatment period (days): 1–6	1.00			1.00		
7–29	0.76	0.52–1.09		0.78	0.52–1.17	
30–89	0.79	0.50–1.25		0.74	0.43–1.27	
90 or more	0.95	0.43–2.09		1.01	0.41–2.48	
Number of previous hospitalizations: 0	1.00			1.00		
1	0.82	0.51–1.31		0.79	0.48–1.28	
2	2.44	1.44–4.13		2.55	1.47–4.42	
3 or more	3.40	2.24–5.17		3.35	2.15–5.23	
Exposure to ward overload during index treatment period: <20% of days	1.00			1.00		
20–80% of days	0.74	0.51–1.07		0.89	0.59–1.37	
> 80% of days	0.78	0.51–1.18		0.80	0.51–1.25	

^a^All variables entered simultaneously

OR = odds ratio; CI = confidence interval

Supplementary Table 2 presents the results from the models in which only individual-level factors and individual plus treatment factors were entered. The findings are largely in accordance with the main two models presented in Tables 1, 2 and 3.

### Interaction analyses

We did not find any interactions between ward overload and psychiatric comorbidity predicting readmission (30 days, one year, or multiple readmissions; *p*-values > 0.10). Regarding sex, there was a significant interaction with psychotic disorder predicting readmission within 30 days (*p* = 0.011). When the analyses were conducted separately for women and men, the likelihood of readmission among women with a psychotic disorder was 0.68 (95% CI 0.44–1.05, adjusted for age). The corresponding likelihood among men was 1.29 (0.83–2.02).

## Discussion

In this prospective study, we focused on psychiatric readmissions in one hospital district in Finland. From the available administrative and clinical data, we examined the associations of patient-, treatment- and ward-level factors with readmission. The most prevalent diagnoses were psychotic disorders (43%) and depressive and other mood disorders (35%). Approximately one-third of the treatment periods lasted less than one week. Readmission was relatively common: 11% of the patients returned within 30 days, and a third returned within a year. These figures are in line with previous studies from Finland [5].

There were slightly more women (52%) than men (48%), which is in accordance with a previous national study [5]. There were no major differences in readmission between women and men; however, men were less likely than women to have a readmission within 30 days. A previous study suggested no difference between women and men in Finland [3]. In general, clinical and biopsychosocial factors may explain the difference between women and men. Women have a greater tendency to seek treatment when experiencing symptoms [21]. It has also been suggested that cultural assumptions related to both gender and health are produced in social interaction with the environment; therefore, gender roles might guide treatment seeking among women and men [22]. However, the interaction analysis suggested that women with a psychotic disorder might have a lower likelihood of readmission within 30 days than women without a psychotic disorder. As this association was not statistically significant, this question remains to be examined in future studies.

We found that in some models, those in the oldest age group (> 70 years) had a lower likelihood of readmission within a year while no association with age was found for 30-day readmission or multiple readmissions. This is in line with previous studies carried out in an adult population in Finland [3] and internationally [10, 14, 18], in which younger patients have been more likely to return to the hospital than older patients. However, we also found that patients with disorders of psychological development and behavioural and emotional disorders with onset usually occurring in childhood and adolescence were less likely to experience readmission within 30 days. In addition, frequent readmissions were also less likely in this group, where the patients typically are children and adolescents. Our findings may reflect the effort to implement treatment in outpatient care whenever possible so that the child’s or young person’s connection to the family and other support networks is not broken. However, it is important to acknowledge that in child and adolescent psychiatry, there has been a shortage of resources for years, which can mean that you cannot reach a hospital even if there is a need.

We found that neurotic, stress-related and somatoform disorders were associated with readmission within 30 days. The examination of neurotic, stress-related and somatoform disorders is new, as previous research has mostly focused on severe mental disorders and depression. The few studies including these disorders have suggested mixed findings [10]. Many stress-related and anxiety disorders, such as posttraumatic stress disorder (PTSD), are relatively severe; however, generalized anxiety disorder (GAD) is considered a milder disorder. PTSD can be long-lasting and requires intensive treatment. Typical symptoms of GAD include excessive worry about one’s own symptoms, which can increase help-seeking behaviour.

Psychotic disorders, such as schizophrenia, were associated with readmission within one year. Schizophrenia has been associated with the likelihood of readmission in previous studies [3, 9–11]. This reflects the severity of psychotic disorders and the need for inpatient treatment for these disorders.

Having a behavioural syndrome associated with physiological disturbances and physical factors or disorders of adult personality and behaviour was associated with readmission. The few previous studies on personality disorders have suggested an association with readmissions [10] whereas we are not aware of previous studies on eating disorders. In the present study, eating disorders were the most prevalent disorders in this group of mental disorders. Eating disorders include anorexia nervosa, bulimia nervosa and binge eating disorder. The most severe, complex, and long-lasting cases of eating disorders are treated in specialized hospital care [23]. Approximately 70% of the patients recover, but only a quarter recover within a year, which suggests a long-lasting recovery process with several readmissions [23]. Of the personality disorders, the prognosis of patients with emotionally unstable personality disorders is quite good; more than half of them no longer fulfil the diagnostic criteria after five years [24]. However, this and other personality disorders, such as paranoid, schizoid, dissocial, anxious (avoidant), or dependent personality can weaken the treatment efficacy for other disorders, such as depression [25]. All diagnoses were recorded in our dataset, so it is possible that personality disorders were coded as secondary diagnoses.

Comorbid mental disorders were neither directly associated with readmissions nor was there any interaction between comorbidity and ward overload as a predictor of readmission. The lack of evidence is in line with previous research which suggests that the association between psychiatric comorbidity and readmission is inconclusive [10]. Comorbid somatic disease was not associated with readmission either, which is in contrast with previous findings showing an increased risk of readmission associated with somatic comorbidity [3, 13, 15]. In our study, a small negative association was found only within one year. It is possible that somatic comorbidity can play a role, for example, in older patient groups, which could be examined with larger datasets.

Neuromodulation treatment was associated with readmission within 30 days, and among those with at least one readmission, it was associated with frequent readmissions. In a previous study with a Chinese sample, ECT treatment was associated with a lower likelihood of readmission [14]. However, in China, ECT treatment is also provided for treatment groups other than those with depression, e.g., for those with schizophrenia, and in that study, the effect of ECT on readmission was shown among schizophrenia patients [14]. In our study, readmission can be related to planned readmissions; the treatment procedure included new, agreed-upon neuromodulation sessions.

In line with previous findings [10, 11], we found that a history of psychiatric hospitalizations was consistently associated with readmission and frequent readmissions. Having hospitalizations in the past may be a proxy for several disease-, patient- and environmental-related factors affecting hospitalization for mental disorders. However, the length of the index period was not associated with readmission. A systematic review suggested mixed findings on this issue [10]. The authors concluded that among those with schizophrenia or substance use disorders, longer inpatient treatment could protect against relapse. However, future research could examine in more detail the role the duration of treatment plays in different diagnostic groups. Some studies have applied natural experimental designs to assess the impacts of system-level decisions to shorten hospital treatments. These studies have found that readmissions increased after the shortening of treatment periods [12].

We did not find associations with ward overload, as expressed by the bed occupancy rate. Previous studies focusing on hospital capacity have usually utilized the number of patients or the number of beds to represent hospital capacity rather than bed occupancy, as in our study, and the findings have been mixed [9, 12]. Other studies have focused on personnel resources, and in those studies, better resources in the hospital district [9] or unit [16, 17] were associated with a lower risk of readmission, although in one study, the area-level number of mental healthcare personnel per inhabitant was not associated with readmission [15]. Overload in the hospital ward can have many effects on the treatment process. For example, in long-term overloaded wards, access to treatment can become more difficult. This might have had an impact on our results, as the difficulties in admissions may have led to fewer readmissions in our patient population. In further studies, it would be important to examine the link between ward overload and access to acute psychiatric inpatient care.

The strengths of our study include the use of large, routinely collected data from an entire hospital district, which enabled us to examine readmissions in detail. Restricting the study to one hospital district excluded the possibility of regional differences affecting the findings [5]. However, there are also several limitations. Since the data available for this study were limited to inpatient care records, we had not, for example, the possibility to consider factors that were related to ward personnel, such as nurse-to-patient ratio [16, 17] and to assess post-discharge characteristics. The quantity and quality of outpatient services after inpatient treatment are important topics for future research [26], as a previous study in Finland showed that those without outpatient treatment contact were at the greatest risk of readmission [5]. For some hospital wards, the ward overload variable covered more than one sub-unit which may had led to some inaccuracy in the estimates. Our study did not assess several patient characteristics, such as disorder-specific factors, commitment to treatment, and economic circumstances [26]. The prevalence of somatic diseases was 10%, which suggests that they are under recorded in psychiatric medical records. Therefore, we did not conduct interaction analyses between psychiatric and somatic diseases. This could be an important topic for future studies [27]. Although we did not find any meaningful interactions between the assessed factors, future studies could continue this line of research by identifying vulnerable groups. In addition, we did not have information on whether the readmission was planned. In Finland, approximately 80% of readmissions are unplanned [5]. Finally, our findings are generalizable to one hospital district in Finland.

## Conclusions

The findings of this study provide new information on the factors related to readmission in psychiatric inpatient care, such as several rarely explored diagnostic groups, comorbidity, the number of readmissions, ward overload as a potential contextual factor for readmission. Furthermore, we explored the potential interaction effects between specific predictors. Women, patients with specific diagnoses and those with previous hospitalizations may have an increased risk of readmission. The risk factors were somewhat different for single and multiple readmissions; specific sociodemographic factors and diagnostic groups emerged in single readmissions while for repeated readmission, only development and childhood -related diagnoses predicted lower occurrence while a history of psychiatric treatment predicted higher occurrence of repeated readmission. In the future, larger studies could examine whether these findings are generalizable to the entire country and other countries. Readmission is considered an important indicator of the quality of care [4]. Regarding children and young people, it is important to examine whether access to treatment is realized according to their needs.

## Electronic supplementary material

Below is the link to the electronic supplementary material.

Supplementary Material 1

## Data Availability

The data used in this study were obtained from the Auria Clinical Informatics database and were not publicly available. Contact information: https://www.auria.fi/tietopalvelu/en/index.html.

## Abbreviations

CI: Confidence interval
ECT: Electroconvulsive therapy
GAD: Generalized anxiety disorder
ICD-10: International Classification of Diseases, 10th Revision
OR: Odds ratio
PTSD: Posttraumatic stress disorder

## References

[1] Linnaranta O. Report on the number of psychiatric inpatient beds [in Finnish]. Helsinki: Finnish Institute for Health and Welfare; 2022.

[2] Linnaranta O. Recommendations to ensure adequate and high-quality psychiatric hospital care [in Finnish]. Helsinki: Finnish Institute for Health and Welfare; 2022.

[3] Katschnig H, Straßmayra C, Endela F, Berger M, Zauner G, kalseth J, et al. Using national electronic health care registries for comparing the riskof psychiatric re-hospitalisation in six European countries: opportunities and limitations. Health Policy. 2019;123:1028–35. 10.1016/j.healthpol.2019.07.006 31405616 10.1016/j.healthpol.2019.07.006

[4] Wahlbeck K, Haaramo P, Cresswell-Smith J. Development of mental health services: utilization of register data on psychiatric care [in Finnish]. Helsinki: Finnish Institute for Health and Welfare; 2017.

[5] Wahlbeck K, Cresswell-Smith J, Haaramo P. Readmission to psychiatric care. Regional incidence and associations with outpatient care [in Finnish]. Finnish Med J. 2019;74:120–24.

[6] Ådnanes M, Melby L, Cresswell-Smith J, Westerlund H, Rabbi L, Dernovsek MZ, et al. Mental health service users’ experiences of psychiatric re-hospitalisation - an explorative focus group study in six European countries. BMC Health Serv Res. 2018;18:516. 10.1186/s12913-018-3317-1 29970098 10.1186/s12913-018-3317-1 PMC6029175

[7] Chi MH, Hsiao CY, Chen KC, Lee L-T, Tsai HC, Lee IH, et al. The readmission rate and medical cost of patients with schizophrenia after first hospitalization — a 10-year follow-up population-based study. Schizophrenia Res. 2016;170:184–90. 10.1016/j.schres.2015.11.025 10.1016/j.schres.2015.11.025 26678982

[8] Wood L, Alsawy S. Patient experiences of psychiatric inpatient care: a systematic review of qualitative evidence. J Psychiatr Int Care. 2016;12:35–43. 10.20299/jpi.2016.001

[9] Tedeschi F, Donisi V, Salazzari D, Cresswell-Smith J, Wahlbeck K, Amaddeo F. Clinical and organizational factors predicting readmission for mental health patients across Italy. Soc Psychiatry Psychiatr Epidemiol. 2020;55:187–96. 10.1007/s00127-019-01766-y 31463615 10.1007/s00127-019-01766-y

[10] Donisi V, Tedeschi F, Wahlbeck K, Haaramo P, Amaddeo F. Pre-discharge factors predicting readmissions of psychiatric patients: a systematic review of the literature. BMC Psychiatry. 2016;16:449. 10.1186/s12888-016-1114-0 27986079 10.1186/s12888-016-1114-0 PMC5162092

[11] Machado V, Leonidas C, Santos MA, Souza J. Psychiatric readmission: an integrative review of the literature. Int Nurs Rev. 2012;59:447–57. 10.1111/j.1466-7657.2012.01011.x 10.1111/j.1466-7657.2012.01011.x 23134127

[12] Kalseth J, Lassemo E, Wahlbeck K, Haaramo P, Magnussen J. Psychiatric readmissions and their association with environmental and health system characteristics: a systematic review of the literature. BMC Psychiatry. 2016;16:376. 10.1186/s12888-016-1099-8 27821155 10.1186/s12888-016-1099-8 PMC5100223

[13] Šprah L, Dernovšek M, Wahlbeck K, Haaramo P. Psychiatric readmissions and their association with physical comorbidity: a systematic literature review. BMC Psychiatry. 2017;17:2. 10.1186/s12888-016-1172-3 28049441 10.1186/s12888-016-1172-3 PMC5210297

[14] Han X, Jiang F, Tang Y, Needleman J, Guo M, Chen Y, et al. Factors associated with 30-day and 1-year readmission among psychiatric inpatients in Beijing China: a retrospective, medical record-based analysis. BMC Psychiatry. 2020;20:113. 10.1186/s12888-020-02515-1 32160906 10.1186/s12888-020-02515-1 PMC7065326

[15] Mascayano F, Haselden M, Corbeil T, Wall MM, Tang F, Essock SM, et al. Patient-, hospital-, and system-level factors associated with 30-day readmission after a psychiatric hospitalization. J Nerv Ment Dis. 2022;210:741–6. 10.1097/NMD.0000000000001529 35472041 10.1097/NMD.0000000000001529

[16] Okumura Y, Sugiyama N, Noda T, Sakata N. Association of high psychiatrist staffing with prolonged hospitalization, follow-up visits, and readmission in acute psychiatric units: a retrospective cohort study using a nationwide claims database. Neuropsychiatr Dis Treat. 2018;14:893–902. 10.2147/NDT.S160176 29636614 10.2147/NDT.S160176 PMC5880415

[17] Park S, Park S, Lee YJ, Choon-Seon P, Young-Chul J, Sunah K. Nurse staffing and health outcomes of psychiatric inpatients: a secondary analysis of national health insurance claims data. J Korean Acad Nurs. 2020;50:333–48. 10.4040/jkan.19203 32632069 10.4040/jkan.19203

[18] Berardelli I, Sarubbi S, Rogante E, Erbuto D, Cifrodelli M, Giuliani C, et al. Exploring risk factors for re-hospitalization in a psychiatric inpatient setting: a retrospective naturalistic study. BMC Psychiatry. 2022;22:821. 10.1186/s12888-022-04472-3 36550540 10.1186/s12888-022-04472-3 PMC9783999

[19] Fogg C, Bridges J, Meredith P, Spice C, Field L, Culliford D. The association between ward staffing levels, mortality and hospital readmission in older hospitalised adults, according to presence of cognitive impairment: a retrospective cohort study. Age Ageing. 2021;50:431–9. 10.1093/ageing/afaa133 32970798 10.1093/ageing/afaa133

[20] Daud-Gallotti RM, Costa SF, Guimaraes T, Padilha KG, Inoue EN, Vasconcelos TN, et al. Nursing workload as a risk factor for healthcare associated infections in ICU: a prospective study. PLoS ONE. 2012;7:e52342. 10.1371/journal.pone.0052342 23300645 10.1371/journal.pone.0052342 PMC3531467

[21] Muka T, Imo D, Jaspers L, Colpani V, Chaker L, van der Lee SJ, et al. The global impact of non-communicable diseases on healthcare spending and national income: a systematic review. Eur J Epidemiol. 2015;30:251–77. 10.1007/s10654-014-9984-2 25595318 10.1007/s10654-014-9984-2

[22] Pattyn E, Verhaeghe M, Bracke P. The gender gap in mental health service use. Soc Psychiatry Psychiatr Epidemiol. 2015;50:1089–95. 10.1007/s00127-015-1038-x. 25788391 10.1007/s00127-015-1038-x

[23] Keski-Rahkonen A. Eating disorders – temporary annoyance or eternal trouble? [in Finnish] Duodecim. 2010;126:2209–14. 21072967

[24] Treatment guidelines. Unstable personality. Duodecim; 2020 [In Finnish, available at: https://www.kaypahoito.fi/en/]

[25] Young M. Prognostic significance of personality disorders in patients with major depressive disorder. Curr Treat Options Psych. 2020;7:559–69. 10.1007/s40501-020-00227-7

[26] Sfetcu R, Musat S, Haaramo P, Ciutan M, Scintee G, Vladescu C, et al. Overview of post-discharge predictors for psychiatric re-hospitalisations: a systematic review of the literature. BMC Psychiatry. 2017;17:227. 10.1186/s12888-017-1386-z 28646857 10.1186/s12888-017-1386-z PMC5483311

[27] Kavalidou K, Smith DJ, Der G, O’Connor RC. The role of physical and mental multimorbidity in suicidal thoughts and behaviours in a Scottish population cohort study. BMC Psychiatry. 2019;19:1–9. 30674288 10.1186/s12888-019-2032-8 PMC6344985

