# Supplementary material for: Factors associated with readmissions in psychiatric inpatient care: a prospective cohort study based on hospital registers
Source: BMC Psychiatry. 2024 Oct 25;24:734. doi: 10.1186/s12888-024-06193-1 (PMC11515830; doi:10.1186/s12888-024-06193-1)
Supplement: Supplementary file 1 — Supplementary Material 1 [file 12888_2024_6193_MOESM1_ESM.docx]

| **Supplementary Table 1** Characteristics of the patients (n=2052) | |
| --- | --- |
|  | % |
| Sex: female | 52.1 |
| Male | 47.9 |
| Age group: 0–15 | 11.5 |
| 16–30 | 33.4 |
| 31–50 | 26.8 |
| 51–70 | 18.3 |
| 71– | 10.0 |
| Mental and behavioural disorders due to psychoactive substance use (F10–F19): no | 84.2 |
| yes | 15.8 |
| Psychotic disorders; Schizophrenia. schizotypal and delusional disorders (F20–F29), manic episode, bipolar affective disorder (F30–F31): no | 56.7 |
| yes | 43.3 |
| Depressive disorders and other mood disorders (F32–F39): no | 65.0 |
| yes | 35.0 |
| Neurotic. stress-related and somatoform disorders (F40–F48): no | 80.0 |
| yes | 20.0 |
| Behavioural syndromes associated with physiological disturbances and physical factors (F50–F59); disorders of adult personality and behaviour (F60–F69): no | 89.3 |
| yes | 10.7 |
| Disorders of psychological development (F80–F89); behavioural and emotional disorders with onset usually occurring in childhood and adolescence (F90–F98): no | 90.1 |
| yes | 9.9 |
| Number of mental disorders in the examined categories: 0-1^a^ | 68.3 |
| 2 or more | 31.7 |
| Somatic disease: no | 90.0 |
| yes | 10.0 |
| Neuromodulation treatment: no | 97.8 |
| yes | 2.2 |
| Length of index treatment period (days): 1-6 | 27.7 |
| 7–29 | 50.1 |
| 30–89 | 18.6 |
| 90 or more | 3.6 |
| Number of previous hospitalizations since 2008: 0 | 64.2 |
| 1 | 15.3 |
| 2 | 7.0 |
| 3 or more | 13.5 |
| Exposure to ward overload during index treatment period: <20% of days | 28.1 |
| 20–80% of days | 42.8 |
| >80% of days | 29.1 |

^a^n=86 did not have a recorded mental disorder.

**Supplementary Table 2.** Logistic regression analysis for all readmission outcomes, with models that include individual-level and individual + treatment factors

|  | **Readmission within 30 days** | | | | |  | **Readmission within a year** | | | | |  | **Multiple readmissions versus one** | | | | | |
| --- | --- | --- | --- | --- | --- | --- | --- | --- | --- | --- | --- | --- | --- | --- | --- | --- | --- | --- |
| Characteristics | Individual factors | | | Individual + treatment | | | Individual factors | | | Individual + treatment | | | Individual factors | | | Individual + treatment | | |
|  | OR | 95% CI | | OR | 95% CI | | OR | 95% CI | | OR | 95% CI | | OR | 95% CI | | OR  95% CI | |  |
| Sex |  |  |  |  |  |  |  |  |  |  |  |  |  |  |  |  |  |  |
| Women | 1.00 |  |  | 1.00 |  |  | 1.00 |  |  | 1.00 |  |  | 1.00 |  |  | 1.00 |  |  |
| Men | 0.99 | 0.74 | 1.34 | 1.00 | 0.74 | 1.36 | **0.80** | **0.66** | **0.98** | **0.80** | **0.66** | **0.98** | 0.88 | 0.63 | 1.22 | 0.88 | 0.62 | 1.25 |
| Age group |  |  |  |  |  |  |  |  |  |  |  |  |  |  |  |  |  |  |
| < 16 | 1.00 |  |  | 1.00 |  |  | 1.00 |  |  | 1.00 |  |  | 1.00 |  |  | 1.00 |  |  |
| 16-30 | 0.97 | 0.56 | 1.67 | 0.89 | 0.51 | 1.54 | 1.02 | 0.71 | 1.48 | 0.94 | 0.65 | 1.37 | 0.95 | 0.51 | 1.77 | 0.72 | 0.38 | 1.36 |
| 31-50 | 0.73 | 0.40 | 1.31 | 0.65 | 0.36 | 1.19 | 0.83 | 0.56 | 1.24 | 0.74 | 0.49 | 1.10 | 1.02 | 0.53 | 1.96 | 0.74 | 0.37 | 1.47 |
| 51-70 | 0.92 | 0.50 | 1.69 | 0.81 | 0.44 | 1.51 | 0.92 | 0.60 | 1.39 | 0.79 | 0.51 | 1.21 | 1.03 | 0.51 | 2.06 | 0.72 | 0.35 | 1.48 |
| 71+ years | 0.74 | 0.37 | 1.49 | 0.68 | 0.34 | 1.38 | **0.58** | **0.35** | **0.94** | **0.53** | **0.32** | **0.87** | 0.76 | 0.32 | 1.78 | 0.49 | 0.19 | 1.22 |
| Mental and behavioural disorders due to psychoactive substance use (F10–F19) | | | | | | | | |  |  |  |  |  |  |  |  |  |  |
| No | 1.00 |  |  | 1.00 |  |  | 1.00 |  |  | 1.00 |  |  | 1.00 |  |  | 1.00 |  |  |
| Yes | 1.03 | 0.58 | 1.80 | 1.01 | 0.57 | 1.78 | 1.30 | 0.89 | 1.89 | 1.34 | 0.92 | 1.97 | 0.96 | 0.54 | 1.70 | 1.04 | 0.57 | 1.91 |
| Psychotic disorders; Schizophrenia, schizotypal and delusional disorders (F20–F29); manic episode, bipolar affective disorder (F30–F31) | | | | | | | | | | | | | | | | | |  |
| No | 1.00 |  |  | 1.00 |  |  | 1.00 |  |  | 1.00 |  |  | 1.00 |  |  | 1.00 |  |  |
| Yes | 1.40 | 0.84 | 2.35 | 1.45 | 0.86 | 2.46 | **1.59** | **1.11** | **2.26** | **1.46** | **1.02** | **2.11** | 0.83 | 0.47 | 1.45 | 0.86 | 0.47 | 1.58 |
| Depressive disorders and other mood disorders (F32–F39) | | | | |  |  |  |  |  |  |  |  |  |  |  |  |  |  |
| No | 1.00 |  |  | 1.00 |  |  | 1.00 |  |  | 1.00 |  |  | 1.00 |  |  | 1.00 |  |  |
| Yes | 1.56 | 0.93 | 2.60 | 1.59 | 0.95 | 2.67 | 1.17 | 0.82 | 1.66 | 1.18 | 0.82 | 1.69 | 0.75 | 0.43 | 1.32 | 0.71 | 0.39 | 1.30 |
| Neurotic, stress-related and somatoform disorders (F40–F48) | | | | | |  |  |  |  |  |  |  |  |  |  |  |  |  |
| No | 1.00 |  |  | 1.00 |  |  | 1.00 |  |  | 1.00 |  |  | 1.00 |  |  | 1.00 |  |  |
| Yes | **1.93** | **1.13** | **3.30** | **2.08** | **1.21** | **3.59** | 1.33 | 0.91 | 1.94 | 1.41 | 0.96 | 2.08 | 0.89 | 0.49 | 1.60 | 1.07 | 0.57 | 1.99 |
| Behavioural syndromes associated with physiological disturbances and physical factors (F50–F59), disorders of adult personality and behaviour (F60–F69) | | | | | | | | | | | | | | | | | | |
| No | 1.00 |  |  | 1.00 |  |  | 1.00 |  |  | 1.00 |  |  | 1.00 |  |  | 1.00 |  |  |
| Yes | **1.95** | **1.12** | **3.42** | **1.87** | **1.06** | **3.31** | **2.03** | **1.37** | **3.00** | **1.80** | **1.21** | **2.69** | 1.27 | 0.71 | 2.27 | 1.14 | 0.61 | 2.11 |
| Disorders of psychological development (F80–F89); behavioural and emotional disorders with onset usually occurring in childhood and adolescence (F90–F98) | | | | | | | | | | | | | | | | | | |
| No | 1.00 |  |  | 1.00 |  |  | 1.00 |  |  | 1.00 |  |  | 1.00 |  |  | 1.00 |  |  |
| Yes | 0.62 | 0.29 | 1.32 | 0.59 | 0.27 | 1.26 | 1.09 | 0.69 | 1.70 | 0.99 | 0.63 | 1.57 | **0.44** | **0.21** | **0.94** | **0.36** | **0.16** | **0.80** |
| Number of mental disorders in the examined categories |  |  |  |  |  |  |  |  |  |  |  |  |  |  |  |  |  |  |
| 0-1 | 1.00 |  |  | 1.00 |  |  | 1.00 |  |  | 1.00 |  |  | 1.00 |  |  | 1.00 |  |  |
| 2+ | 0.54 | 0.28 | 1.02 | **0.51** | **0.27** | **0.98** | 0.70 | 0.45 | 1.07 | 0.67 | 0.43 | 1.04 | 1.31 | 0.67 | 2.58 | 1.25 | 0.61 | 2.54 |
| Somatic disease |  |  |  |  |  |  |  |  |  |  |  |  |  |  |  |  |  |  |
| No | 1.00 |  |  | 1.00 |  |  | 1.00 |  |  | 1.00 |  |  | 1.00 |  |  | 1.00 |  |  |
| Yes | 0.96 | 0.59 | 1.57 | 0.99 | 0.61 | 1.63 | 0.71 | 0.51 | 1.01 | 0.73 | 0.51 | 1.03 | 1.13 | 0.63 | 2.05 | 1.12 | 0.60 | 2.09 |
| Neuromodulation treatment |  |  |  |  |  |  |  |  |  |  |  |  |  |  |  |  |  |  |
| No |  |  |  | 1.00 |  |  |  |  |  | 1.00 |  |  |  |  |  | 1.00 |  |  |
| Yes |  |  |  | **2.40** | **1.08** | **5.34** |  |  |  | **1.64** | **0.87** | **3.08** |  |  |  | **5.38** | **1.74** | **16.63** |
| Length of index treatment period (days) | |  |  |  |  |  |  |  |  |  |  |  |  |  |  |  |  |  |
| 1-6 |  |  |  | 1.00 |  |  |  |  |  | 1.00 |  |  |  |  |  | 1.00 |  |  |
| 7-29 |  |  |  | 0.88 | 0.63 | 1.22 |  |  |  | 1.04 | 0.83 | 1.32 |  |  |  | 0.78 | 0.53 | 1.17 |
| 30-89 |  |  |  | **0.62** | **0.39** | **0.99** |  |  |  | 1.11 | 0.83 | 1.50 |  |  |  | 0.74 | 0.44 | 1.23 |
| 90+ |  |  |  | 0.53 | 0.21 | 1.31 |  |  |  | 1.22 | 0.72 | 2.07 |  |  |  | 1.01 | 0.43 | 2.40 |
| Number of previous hospitalizations |  |  |  |  |  |  |  |  |  |  |  |  |  |  |  |  |  |  |
| 0 |  |  |  | 1.00 |  |  |  |  |  | 1.00 |  |  |  |  |  | 1.00 |  |  |
| 1 |  |  |  | 1.45 | 0.97 | 2.17 |  |  |  | **1.39** | **1.06** | **1.82** |  |  |  | 0.79 | 0.49 | 1.29 |
| 2 |  |  |  | **2.06** | **1.26** | **3.36** |  |  |  | **2.27** | **1.59** | **3.25** |  |  |  | **2.54** | **1.47** | **4.40** |
| 3 or more |  |  |  | **1.59** | **1.05** | **2.40** |  |  |  | **2.27** | **1.72** | **3.00** |  |  |  | **3.37** | **2.17** | **5.24** |
